# Real-time photoacoustic imaging with freehand light delivery and augmented reality guidance^☆^

**DOI:** 10.1016/j.pacs.2026.100827

**Published:** 2026-04-28

**Authors:** Junhao Zhang, Cynthia Li, Muyinatu A. Lediju Bell

**Affiliations:** a Department of Electrical and Computer Engineering, Johns Hopkins University, Baltimore, 21218, MD, USA; b Department of Electrical Engineering & Computer Science, Massachusetts Institute of Technology, Cambridge, 02139, MA, USA; c Department of Biomedical Engineering, Johns Hopkins University, Baltimore, 21218, MD, USA; d Department of Computer Science, Johns Hopkins University, Baltimore, 21218, MD, USA

**Keywords:** Augmented reality, Virtual reality, Photoacoustic imaging, Surgical guidance

## Abstract

Photoacoustic-guided surgery benefits from precise co-alignment between detached light delivery and sound reception components. Misalignment can result in absent photoacoustic signals, potentially leading to critical structures incorrectly interpreted as absent. Augmented reality has the potential to provide real-time spatial registration and intuitive visualization through tracking of the laser and ultrasound transducer positions. We developed and validated a novel augmented reality guidance system that tracks a freehand light source and ultrasound transducer, rendering real-time virtual representations of the associated laser beam and ultrasound image to facilitate intuitive alignment. Quantitative evaluation demonstrated a 58% reduction in mean targeting error during a static alignment task, relative to a standard freehand approach implemented without augmented reality. The total variance of laser-image intersection distribution with augmented reality was 12.0 mm${}^{2}$, reduced from 45.9 mm${}^{2}$ without augmented reality, indicating higher motion stability. In addition, the system successfully tracked complex motions, and photoacoustic signals from a blood vessel target hidden by tissue were spatially rendered on the tissue surface to demonstrate surgical guidance capabilities. This work establishes augmented reality as a promising approach to address anticipated challenges surrounding photoacoustic-guided surgery with light delivery separated from acoustic receivers.

## 1 Introduction

Photoacoustic imaging is a promising biomedical modality that combines the high contrast of optical imaging with the deep tissue penetration of ultrasound [1]. By exciting tissue with short-pulsed, non-ionizing laser light and detecting the resulting ultrasonic waves, photoacoustic imaging can map hemoglobin concentration, lipid distribution, and other chromophores in real time with greater contrast than traditional ultrasound [2], [3], [4], [5]. This capability makes photoacoustic imaging particularly valuable for surgical guidance by identifying critical structures such as blood vessels [6], nerves [7], tumors [8], cancellous or cortical bone [9], [10], and contrast-agent-filled ureters [11].

**Fig. 1:**
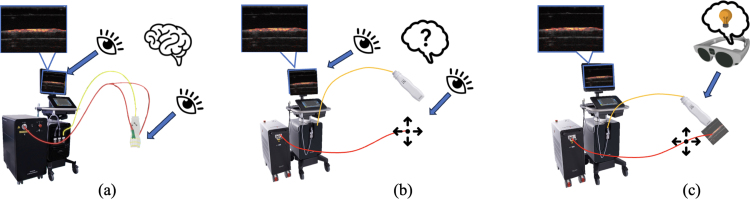
Photoacoustic imaging system configurations: (a) coupled ultrasound transducer and light source, producing a limited region of optimal photoacoustic signal; (b) detached ultrasound transducer and light source, enabling a customizable illumination region at the cost of more challenging tracking; and (c) detached freehand light source with augmented reality guidance.

When photoacoustic imaging is used to guide surgeries, light sources may be attached to the ultrasound transducer [12], [13], [14] and surgical tools [15], [16], [17], [18], or undergo freehand operation [11], [19], [20], to make decisions about the location of critical structures hidden by tissue. However, the fixed coupling between the ultrasound transducer and laser source may limit effective light delivery to an intended part of the photoacoustic image (Fig. 1(a)), particularly farther from the transducer. The independence of light delivery from an ultrasound transducer, despite enabling flexible and targeted laser illumination, introduces an alignment challenge (Fig. 1(b)). In particular, although the photoacoustic effect can be generated if the light is delivered interstitially, the laser path must intersect the slice thickness of the two-dimensional image plane (i.e., typically 1–2 mm in the elevation dimension) of the ultrasound transducer to generate detectable and spatially accurate photoacoustic signals. Misalignment can result in no photoacoustic signals, leading to an incorrect conclusion that there are no critical structures in view, when in reality the existing structure, light profile, and image plane are misaligned [21], [22], [23].

In conventional photoacoustic imaging workflows, operators adjust laser-transducer alignment iteratively based on real-time photoacoustic signal feedback on an external monitor by moving the laser source until maximum signal is observed. This process demands considerable expertise and can be time-consuming during time-sensitive surgical procedures [24]. Augmented reality-guided alignment has the potential to provide capabilities that address limitations of monitor signal-based feedback (Fig. 1(c)).

One of the four key benefits of augmented reality guidance in photoacoustic imaging with freehand light delivery is the potential to enable visualization of the planned illumination geometry before laser activation and photoacoustic signal acquisition. This capability can enable surgeons and other users to optimally position both the laser source and transducer prior to any tissue exposure, which is valuable when planning multi-site imaging protocols or when minimizing laser exposure time is critical.

A second benefit is that augmented reality can render the complete 3D spatial relationship between the laser beam, imaging plane, and anatomical context, enabling surgeons to understand whether or not the current or next alignment is optimal to achieve imaging objectives or otherwise yielding suboptimal photoacoustic signals.

Third, when imaging multiple targets at different locations or performing systematic coverage of a region (e.g., tumor margin assessment), augmented reality facilitates strategic planning of illumination patterns. Signal-based alignment optimizes one location at a time but does not guide systematic spatial coverage strategies.

Fourth, augmented reality provides immediate visual feedback about geometric alignment without requiring repeated laser firing and signal analysis. This capability potentially reduces the number of adjustment iterations needed, which may be important in time-sensitive surgical workflows or when laser exposure should be minimized.

Beyond alignment challenges, widespread adoption of photoacoustic imaging is limited by difficulties in image interpretation and spatial registration. Traditional systems present 2D images on external monitors that require expertise to mentally map to anatomical structures, which can present a distraction during procedures [24]. Without additional guidance, surgeons may need to shift attention from the patient to the image display monitor or ultrasound transducer position, which would increase cognitive load and the potential for errors.

Augmented reality technology offers a compelling solution to surgical guidance challenges by overlaying virtual information onto the physical world viewed by the user. These virtual counterparts to the physical world can be rendered in the original position of objects in the physical world by tracking the positions of the laser and ultrasound transducer, thereby transforming the alignment task from challenging to intuitive [25]. Augmented reality can also be employed to overlay photoacoustic data on the anatomy of a patient, thereby improving spatial understanding and reducing the cognitive burden of mental registration. While augmented reality has been successfully applied to other medical imaging domains, such as ultrasound-guided procedures [26], [27] and computed tomography [28], its integration with photoacoustic imaging is limited.

**Fig. 2:**
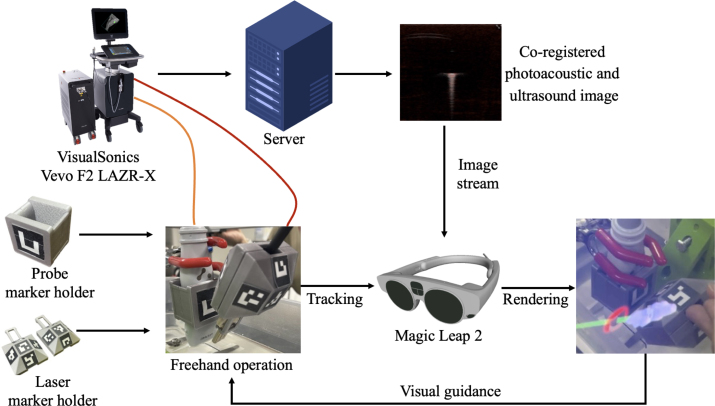
Overview of system architecture for photoacoustic image streaming and device tracking with the Magic Leap 2 augmented reality headset.

Previous work by Suzuki et al. [29] demonstrated a method to overlay preexisting photoacoustic images onto anatomical structures for preoperative visualization. However, this approach only required preoperative photoacoustic images. In addition, this approach [29] and other photoacoustic augmented reality approaches with images provided in real time [30], [31] do not separate the light delivery from acoustic reception nor track the locations of the light source and transducer. Therefore, no existing augmented reality approach addresses the challenges associated with real-time photoacoustic image plane-laser alignment during image acquisitions.

The work herein provides real-time augmented reality guidance for the initial alignment process of the photoacoustic system that will ultimately enable surgeons and other users to efficiently position the laser light and ultrasound transducer to obtain photoacoustic images of targeted structures in real-time. We present the design, implementation, and evaluation of this novel augmented reality-guided photoacoustic imaging system, providing more details than that available in our related conference paper [32]. To enable real-time spatial registration and visualization, our system renders a geometrically calibrated model of the laser beam and overlays a live video feed of the photoacoustic image onto a virtual imaging plane, providing accurate image overlay and intuitive guidance for users.

## 2 Methods

### 2.1 System architecture

The development of the augmented reality assistance system involved three major components: (1) design and fabrication of physical marker holders for the tracked device components, (2) development of a Unity application (Unity Technologies, San Francisco, CA, USA) for the Magic Leap 2 headset (Magic Leap Inc., Plantation, FL, USA) to perform tracking and rendering, and (3) a calibration protocol to ensure the geometric accuracy of the virtual laser beam, as overviewed in Fig. 2. The hardware component of our system was designed to track a handheld laser source (Vevo F2 LAZR-X, FUJIFILM VisualSonics Inc., Toronto, ON, Canada) and ultrasound transducer (UHF29x, FUJIFILM VisualSonics Inc., Toronto, ON, Canada). To facilitate the tracking, custom holders were designed using Autodesk Fusion 360 (Autodesk Inc., San Francisco, CA, USA) to affix ArUco fiducial markers to each device. Our laser holder design incorporated two modular pyramid structures with 20 mm square ArUco markers positioned at 45-degree angles relative to the base, as shown in Fig. 2. This configuration ensured that at least two markers remained visible to the Magic Leap 2 front-facing camera during typical manipulation, mitigating tracking loss due to occlusion. The ultrasound transducer holder was equipped with two 30 mm square markers positioned on opposite sides of the holder, providing robust tracking from a wide range of surgeon viewing angles (Fig. 2).

### 2.2 Coordinate system overview

**Fig. 3:**
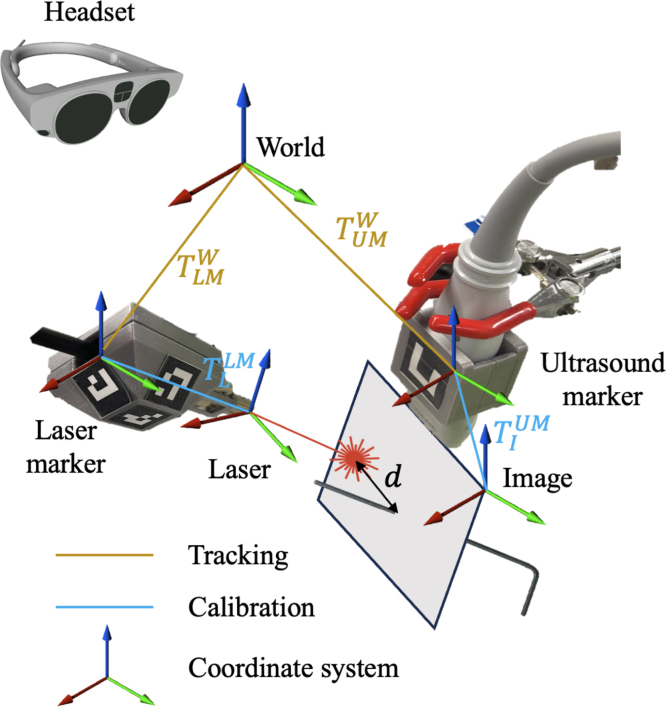
Coordinate systems and transformations.

The spatial coordinate systems and their corresponding transformations are illustrated in Fig. 3. Upon ArUco marker detection, the transformations from the laser marker (LM) and ultrasound marker (UM) coordinate systems to the world coordinate system (W) were obtained as $T_{LM}^{W}$ and $T_{UM}^{W}$, respectively. ArUco markers were rigidly attached to both the laser and the ultrasound transducer, thus the relative transformation between each device and the corresponding ArUco marker remained constant. The transformation from the photoacoustic image (I) to the ultrasound marker (UM), $T_{I}^{UM}$, and the transformation from the laser (L) to the laser marker coordinate systems, $T_{L}^{LM}$, were both derived from the known geometry of the 3D models.

A generic spatial transformation $T_{B}^{A}$ from coordinate system B to A is defined as:

(1) $$T_{B}^{A}\left(p_{B}\right)=p_{A}=q_{B}^{A}\;p_{B}\;{\left(q_{B}^{A}\right)}^{-1}+t_{B}^{A}$$

where $q_{B}^{A}\in S^{3}$ is a unit quaternion, $t\in R^{3}$ is a translation vector, $p_{A}$ and $p_{B}$ are the same point in coordinate systems A and B, respectively. With derived $T_{I}^{UM}$ and tracked $T_{UM}^{W}$, objects in the image coordinate (e.g., pixels) can be visualized by the headset in the world coordinate system.

(2) $$p_{W}=T_{I}^{W}\left(p_{I}\right)=T_{UM}^{W}\cdot T_{I}^{UM}\left(p_{I}\right)$$

Similarly, with derived $T_{L}^{LM}$ and tracked $T_{LM}^{W}$, objects in the laser coordinate (e.g., the virtual laser beam) can be visualized in the world coordinate system.

(3) $$p_{W}=T_{L}^{W}\left(p_{L}\right)=T_{LM}^{W}\cdot T_{L}^{LM}\left(p_{L}\right)$$

### 2.3 Marker detection and pose estimation

The tracking software was implemented as a Unity application with custom C# scripts running on the Magic Leap 2 augmented reality platform. The system utilized markers from the Dictionary_4x4_50 ArUco set, detected through front-facing cameras on the Magic Leap 2 headset. Upon marker detection, the API provided unique marker IDs and associated poses with 6 degrees of freedom (DOF) in the world coordinate system. To determine the device positions and orientations in the world coordinate system after detecting the markers, spatial transformations from the marker origin to the device were computed from 3D models of the device scanned and reconstructed using Polycam software (Polycam Inc., San Francisco, CA, USA). When multiple markers were detected on a single device, the Unity application calculated individual pose candidates by applying stored offsets to the world pose of each marker. To generate a single, stable device pose, the vectors of candidate positions and rotations were averaged using quaternion interpolation.

The averaged pose of the laser beam origin was applied to the virtual counterpart to the physical laser. To mitigate flickering due to transient tracking loss, a 0.1-second buffer was applied, maintaining the virtual object at its last known pose when markers were temporarily undetected. To ensure stable visualization of the image plane and avoid ultrasound transducer tracking noise for accuracy evaluation, the ultrasound transducer was fixed in place, which is a configuration reported in previous photoacoustic guidance systems [16], [17], [33], [34].

The resulting translational calibration error can be estimated based on: (1) the error associated with 3D scanning of the transducer and laser source, denoted as $\sigma_{scan,t}$ (approximately 0.5 mm under ideal conditions [35], [36]); (2) the CAD design alignment error, $\sigma_{CAD,t}$, which is ∼1.0 mm; (3) 3D printing precision or tolerance, $\sigma_{print,t}$, which is ∼0.3 mm, according to the Prusa 3D printer specification [37]; and (4) errors in the physical attachment of the marker holder to the laser source and transducer, $\sigma_{attach,t}$, which is ∼0.5 mm. Assuming these four translational errors are random, independent, isotropic, and linearly accumulated, the total translation error is the square root of the sum of the squares of the individual errors, according to propagation of errors:

(4) $$\sigma_{cal,t}\approx \sqrt{\sigma_{scan,t}^{2}+\sigma_{CAD,t}^{2}+\sigma_{print,t}^{2}+\sigma_{attach,t}^{2}}\approx \sqrt{0.5^{2}+1.0^{2}+0.3^{2}+0.5^{2}}=1.26\;\text{mm}.$$

Based on typical performance of photogrammetry and mechanical assembly tolerances, the rotational calibration error can be estimated as approximately 1–2°.

### 2.4 Kalman filter implementation

To address pose estimation instability and measurement noise, a Kalman filter was implemented to predict and smoothen the 6-DOF pose of each tracked instrument [38]. The filter state was defined as

(5) $$x={\left[x,y,z,\phi ,\theta ,\psi ,v_{x},v_{y},v_{z},\omega_{\phi },\omega_{\theta },\omega_{\psi }\right]}^{T}$$

where x, y, and z are positions, ϕ, θ, and ψ are Euler angles of rotation, and $v_{i}$ and $\omega_{i}$ are the corresponding linear and angular velocities, respectively. A constant-velocity motion model was implemented with the state transition

(6) $$F=\left[\begin{array}{cc} I_{6} & \Delta tI_{6}\\ 0_{6} & I_{6} \end{array}\right]$$

where Δt is the time between the current frame and the previous frame. The process noise covariance Q was set according to the expected variance of positions, rotations, and velocities:

(7) $$Q_{ii}=\left\{\begin{array}{cc} \sigma_{\text{pos}}^{2}\Delta t,\; & i=1,2,3\\ \sigma_{\text{rot}}^{2}\Delta t,\; & i=4,5,6\\ \sigma_{\text{posV}}^{2}\Delta t,\; & i=7,8,9\\ \sigma_{\text{angV}}^{2}\Delta t,\; & i=10,11,12, \end{array}\right)$$

where $\sigma_{\text{pos}}$ and $\sigma_{\text{rot}}$ are the position and rotational noise during hand motion, $\sigma_{\text{posV}}$ and $\sigma_{\text{angV}}$ are the position and rotational velocity noise during hand motion. The off-diagonal elements of the covariance Q were set to zero assuming independent noise between translation and rotation as state components.

The measurement model mapped the state to the observed positions and rotations:

(8) $$z=Hx,\;H=\left[\begin{array}{cccc} I_{3} & 0_{3} & 0_{3} & 0_{3}\\ 0_{3} & I_{3} & 0_{3} & 0_{3} \end{array}\right]$$

The measurement noise was adaptively adjusted based on the number of visible markers. A trust factor T was computed as

(9) $$T=\min\left(\frac{N_{\text{markers}}}{5},1\right)$$

where $N_{\text{markers}}$ is the number of currently visible markers (i.e., five or more visible markers were assigned maximum trust). The effective measurement noise for position $\sigma_{\text{posM,eff}}$ and rotation $\sigma_{\text{rotM,eff}}$ were then scaled according to

(10) $$\sigma_{\text{posM,eff}}=\sigma_{\text{posM}}\left(2-T\right),$$

(11) $$\sigma_{\text{rotM,eff}}=\sigma_{\text{rotM}}\left(2-T\right)$$

where $\sigma_{\text{posM}}$ and $\sigma_{\text{rotM}}$ are the baseline measurement noise values for position and rotation, respectively. These two effective measurement noise values resulted in the diagonal measurement noise covariance matrix R

(12) $$R_{ii}=\left\{\begin{array}{cc} \sigma_{\text{posM,eff}}^{2},\; & i=1,2,3\\ \sigma_{\text{rotM,eff}}^{2},\; & i=4,5,6 \end{array}\right)$$

Fewer visible markers resulted in higher measurement noise, causing the filter to rely more on the predicted motion from the constant-velocity model. The values of our Kalman filter parameters are reported in Table 1. The filtered states were then converted into $T_{UM}^{W}$ and $T_{LM}^{W}$ for visualization of the virtual photoacoustic image and laser beam model, respectively.

To confirm a successful Kalman filter implementation, we performed a controlled linear translation task, moving the laser source along a 17.7-cm path over a 7.8-second time duration, with and without Kalman filtering. The root mean square error of a linear fit to the measured 3D trajectory was 8.03 mm without Kalman filtering and reduced to 1.37 mm with the Kalman filter, due to a reduction of noise, jitter, and unrealistically abrupt pose changes.

**Table 1:** Kalman filter parameters.

Parameter	Description	Values
$\sigma_{\text{pos}}$	Position noise in motion	1 mm
$\sigma_{\text{rot}}$	Rotation noise in motion	0.01 rad
$\sigma_{\text{posV}}$	Velocity noise	0.1 mm/s
$\sigma_{\text{rotV}}$	Rotational velocity noise	0.001 rad/s
$\sigma_{\text{posM}}$	Position noise from tracking	5 mm
$\sigma_{\text{rotM}}$	Rotation noise from tracking	0.05 rad

### 2.5 Visualization and interaction

To render the virtual image plane, a Unity quad object with dimensions of 23.35 mm × 23.0 mm, matching the imaging area of the ultrasound transducer, was created at the beginning of the Unity application. On a server computer running a custom Python script and connected to the photoacoustic imaging system, each photoacoustic image frame was compressed using the Deflate algorithm [39]. The resulting compressed byte stream was then preceded by a 4-byte header indicating its length before being transmitted over TCP to the augmented reality client.

To render the virtual laser beam, the LineRenderer component of the Unity software was utilized. The laser beam is visualized as a transparent cylinder extending from the laser source. This geometric approximation represents the beam axis rather than the actual intensity distribution, which varies with tissue optical properties. Operators use this visualization to align the beam axis with the imaging plane center. A dynamic elliptical visual element represented the virtual laser beam intersection with the virtual photoacoustic imaging plane. Laser-plane intersection was detected using the raycast system of the Unity software. When this intersection occurred, the elliptical visual element, positioned at the intersection point, provided visual feedback for laser-transducer alignment.

The two rendering threads occurred concurrently with data reception on the client side (i.e., augmented reality headset). In particular, a dedicated data reception thread was employed to asynchronously perform data reception, header parsing, frame extraction, then decompression, followed by buffering, to ensure smooth real-time rendering in Unity. The concurrent data reception and rendering multithread operations prevented network and decompression operations from blocking the Unity main rendering thread, thereby allowing continuous frame updates and smooth rendering performance. After decompression, each frame was stored in a buffer queue to minimize latency and limit memory buildup. Each frame was converted into a Texture2D object (640×480, RGB24) and updated at 30 Hz for real-time display, asynchronously from the data reception thread.

**Fig. 4:**
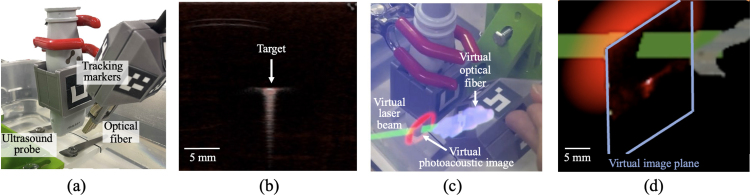
(a) Experimental setup with fixed ultrasound transducer and handheld laser, each tracked with custom ArUco markers. (b) Co-registered photoacoustic and ultrasound images displaying target signal at the imaging plane center. (c) Augmented reality user view showing virtual laser beam (green) intersecting virtual image plane (red) with live photoacoustic image overlay. The virtual laser beam is rendered as a semi-transparent cylinder representing the beam axis. (d) Demonstration of the Euclidean distance from the virtual laser intersection with the virtual image plane to the center of the virtual image plane used to calculate tracking errors.

### 2.6 Image-laser intersection

The photoacoustic image plane is a 2D plane embedded in the 3D photoacoustic image coordinate system. Given a known point $\left(X_{I},Y_{I},Z_{I}\right)$ on the plane and a normal vector (a,b,c), any point $\left(x_{I},y_{I},z_{I}\right)$ lying on the 2D plane satisfies

(13) $$a\left(x_{I}-X_{I}\right)+b\left(y_{I}-Y_{I}\right)+c\left(z_{I}-Y_{I}\right)=0$$

For simplicity, the image coordinate system was chosen such that the normal vector was (0,0,1) and the pivot point was (0,0,0), so that the image plane coincided with the x-y plane.

Although the laser was rendered as a cylinder, it was modeled as a ray vector to compute the laser intersection with the photoacoustic imaging plane. Specifically, the laser is represented as a line originating from $\left(P_{L},Q_{L},R_{L}\right)$ with a unit direction vector (u,v,w):

(14) $$\left(x_{L},y_{L},z_{L}\right)=t\cdot \left(u,v,w\right)+\left(P_{L},Q_{L},R_{L}\right)$$

where t is the distance along the beam and $\left(x_{L},y_{L},z_{L}\right)$ is the end point from distance of t. In this model, $\left(P_{L},Q_{L},R_{L}\right)$ was set to (0,0,0) and the direction (u,v,w) to (0,0,1).

Using these spatial transformations, any point in the laser coordinate system can be transformed to the image coordinate system via

(15) $$p_{I}=T_{L}^{I}\left(p_{L}\right)=T_{IM}^{I}\cdot T_{W}^{IM}\cdot T_{LM}^{W}\cdot T_{L}^{LM}\left(p_{L}\right)$$

Substituting Eqs. (1), (13), and (14) into Eq. (15) yields a linear equation for t, representing the distance the laser beam travels before intersecting the photoacoustic imaging plane. The intersection point in the image frame, $p_{\text{int},I}$, is then recovered using Eq. (14).

### 2.7 Task-based system evaluation

To assess system performance, three alignment tasks were performed as the server computer recorded the laser-plane intersection coordinates on the virtual imaging plane at a 1-Hz frame rate. First, to evaluate steady-state accuracy, static center targeting was implemented by maintaining the laser source at the imaging plane center during three-minute time intervals, with and without assistance from the augmented reality device (performed by co-author J.Z.). Second, to evaluate continuous motion tracking fidelity as a systematic coverage ability (e.g., relevant to margin assessment [8]), a spiral pattern was created by guiding the laser along spiral trajectories from the periphery to the center, then back out toward the periphery with augmented reality assistance (performed by co-author C.L.). Third, to assess discrete positional accuracy relevant to multi-site sampling (e.g., brachytherapy seeds in prostate cancer treatment with transurethral light delivery [40]), four-corner targeting was implemented by sequentially targeting the four imaging plane corners (in addition to the image center) with augmented reality assistance (performed by co-author C.L.).

To generate a point target in each of the three tasks, a hex key submerged in water was used in the photoacoustic imaging setup (Fig. 4(a)). The position of the hex key was adjusted to the image center according to the co-registered ultrasound image (Fig. 4(b)). With augmented reality guidance, the imaging operators aligned the laser path to intersect the hex key during each task (Fig. 4(c)). The static center targeting task was additionally performed by looking at the photoacoustic images without the augmented reality headset.

The system performance in each task was evaluated using a geometric alignment metric that quantifies the spatial relationship between the laser illumination and the photoacoustic imaging plane. This metric measured alignment accuracy, which is a fundamental prerequisite for photoacoustic signal generation invariant of biological tissues and clinical tasks, rather than image quality metrics such as signal-to-noise ratio (SNR), contrast, contrast-to-noise ratio, generalized contrast-to-noise ratio [41]. The Euclidean distance, d, between the virtual laser intersection point and the center of the virtual photoacoustic plane illustrated in Fig. 4(d) was calculated in each frame:

(16) $$d=\sqrt{{\left(p_{int,I}-p_{cen,I}\right)}^{2}},$$

where $p_{int,I}$ is the intersection point computed in Section 2.6, and $p_{cen,I}$ is photoacoustic image center in the image coordinate system. Acquisitions with no laser-image plane intersections were excluded from the distance calculations, with the greatest percentage of exclusions (i.e., 65%) occurring without augmented reality assistance, as reported in Table 2.

**Table 2:** Summary of exclusions from distance calculations during each alignment task indicated, due to no intersection of the laser beam with the image plane.

	Total acquisitions	Number excluded	Percentage excluded
Static (no AR)	212	138	65%
Static (with AR)	185	0	0%
Spiral motion	186	28	15%
Four-corner	172	31	18%

AR = augmented reality.

**Fig. 5:**
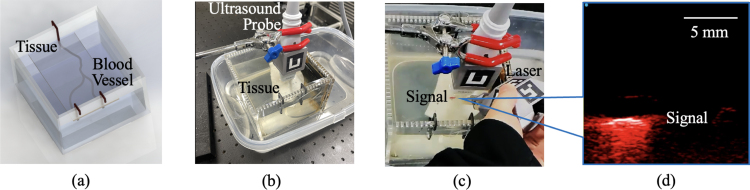
(a) Schematic diagram of the tissue-mimicking validation experiment setup and corresponding photographs (b) with the ultrasound transducer fixed in place and (c) with the freehand laser added and an augmented reality user view showing the photoacoustic real-time photoacoustic signals. (d) Corresponding real-time photoacoustic image as observed on the photoacoustic imaging system monitor.

**Fig. 6:**
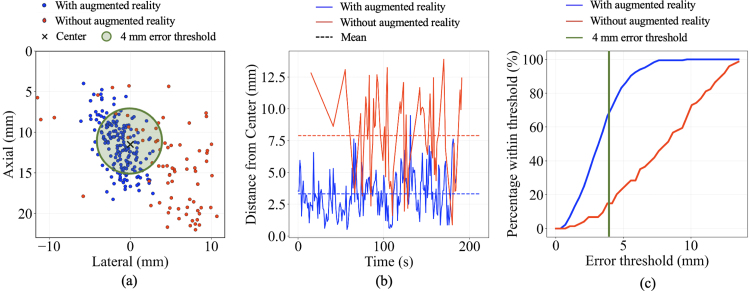
(a) Spatial distribution of laser intersection points on the photoacoustic imaging plane during the static center targeting task with (blue) and without (red) augmented reality guidance. The circle represents an example error threshold of 4 mm surrounding the image plane center, which is represented by the black x. (b) Temporal evolution of distances between laser intersection positions and image center, with the average distances indicated by dashed lines. (c) Percentage of intersections within a specified error threshold, with vertical line indicating the 4 mm error threshold shown in (a).

### 2.8 Validation with *ex vivo* tissue

To demonstrate a potential clinical use of the system, an additional validation was performed with a 3D-printed blood vessel submerged in water, then covered by a ∼1 mm-thick slice of turkey breast. This evaluation is relevant for surgical tasks that require decisions about where to make an incision to avoid vessel injuries, with a concept of the experimental setup shown in Fig. 5(a), a photograph of the actual experiment with the placement of the ultrasound transducer (UHF57x, FUJIFILM VisualSonics Inc., Toronto, ON, Canada) shown in Fig. 5(b), and the addition of the freehand light source shown in Fig. 5(c). The ultrasound transducer differed from that used in prior tasks to demonstrate the versatility of our system design, given the interchangeability of this system component (after updating transformation $T_{I}^{UM}$ shown in Fig. 3).

The user (co-author J.Z.) imaged the hidden vessel with the guidance of virtual photoacoustic signals (shown in Fig. 5(c)), which were processed and rendered from the photoacoustic images displayed on the Visualsonics Vevo F2 monitor, as shown in Fig. 5(d). Real-time image rendering was performed using a custom shader with alpha blending (i.e., pixels with lower amplitudes are more transparent). After identifying the rendered photoacoustic signals, the user locked the rendered signals in position to mark the position of the hidden blood vessel, then the tissue was removed to validate the result by correlating the location of the locked signals with the location of the underlying blood vessel.

## 3 Results

### 3.1 Static center targeting task

Fig. 6 shows results of the static center targeting task (i.e., maintaining the laser at the center of the photoacoustic imaging plane) with an acceptable error threshold (i.e., equidistance from the image plane center) represented as a circle. With the Magic Leap 2 headset (blue data points), the spatial distribution of laser intersection points on the photoacoustic imaging plane (Fig. 6(a)) and the temporal evolution of the targeting errors (Fig. 6(b)) were recorded without access to the Vevo F2 external monitor. When the user relied solely on the Vevo F2 external monitor for visual feedback without augmented reality assistance (red data points), the corresponding results show greater deviations from the target center and acceptable error threshold. In addition, after excluding acquisitions with no laser-image plane intersections, a greater percentage of intersection points resided within a given error threshold (Fig. 6(c)).

Quantitative results for the static centering task are summarized in Table 3. The mean targeting error with augmented reality assistance was 3.31 mm (standard deviation: 1.81 mm), compared to 7.88 mm (standard deviation: 3.31 mm) without augmented reality guidance, representing a 58% reduction in mean targeting error when using the augmented reality system. The total variance of the spatial distribution of laser intersection was 12.0 mm${}^{2}$ and 45.9 mm${}^{2}$ with and without augmented reality, respectively, indicating that the intersection distribution was more concentrated around the mean with augmented reality. The percentages of the laser intersection points within the 4 mm error threshold were 71.9% and 16.2% with and without augmented reality, respectively.

**Table 3:** Summary of performance during the static target centering task, with and without augmented reality assistance.

	With AR	Without AR
Targeting error	3.31 ± 1.81 mm	7.88 ± 3.31 mm
Total variance	12.0 mm${}^{2}$	45.9 mm${}^{2}$
Within 4 mm threshold	71.9%	16.2%

AR = augmented reality.

**Fig. 7:**
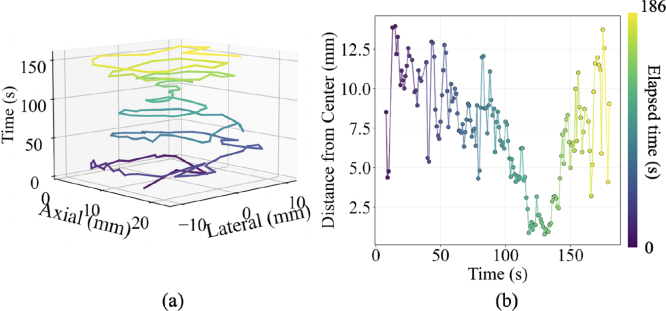
(a) Spatial distribution of laser intersection points on the imaging plane during the spiral motion task. (b) Temporal evolution of distances between laser intersection positions and imaging plane center. The elapsed time is color coded.

### 3.2 Spiral motion task

Fig. 7 shows results of the spiral motion task, demonstrating the ability to track continuous motion with the Magic Leap 2 headset. In Fig. 7(a), the spatial distribution of laser intersection points on the image plane color-coded temporal evolution have a spiral motion initially inward into the center, then outward. In Fig. 7(b), the distance between the intersection point and the image center generally decreases during inward spirals into the center and then increases during outward spirals away from the center. The mean ± one standard deviation of the distance to the image center was 7.2 ± 3.3 mm, which is similar to the targeting error without augmented reality (reported in Table 3), considering that a majority of the image plane was intentionally traversed.

### 3.3 Four-corner targeting task

**Fig. 8:**
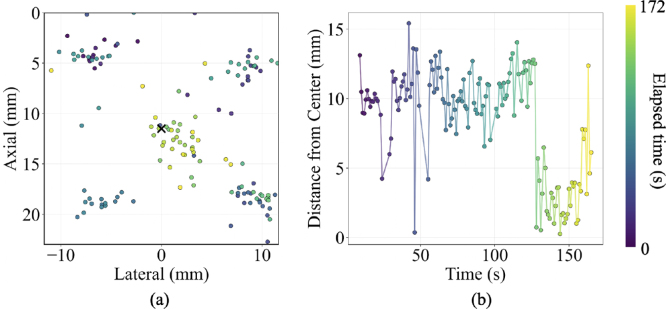
(a) Spatial distribution of laser intersection points on the imaging plane during the four-corner targeting task. The image center is represented by the black x. (b) Temporal evolution of distances between laser intersection positions and imaging plane center. The elapsed time is color coded.

Fig. 8 shows the results of the four-corner targeting task, demonstrating the discrete positional accuracy of the system. In Fig. 8(a), the laser-plane intersection points appear at the four corners, then the image plane center, which corresponds to the temporal trajectory implemented. In Fig. 8(b), measured distances correspond to the expected positions, with higher distances between the corners and the center, transitioning to lower distances at the image center. The mean ± one standard deviation of the distance to the image center was 8.4 ± 3.7 mm, which is comparable to the targeting error without augmented reality (reported in Table 3), considering that corners of the image plane were included in both cases.

**Fig. 9:**
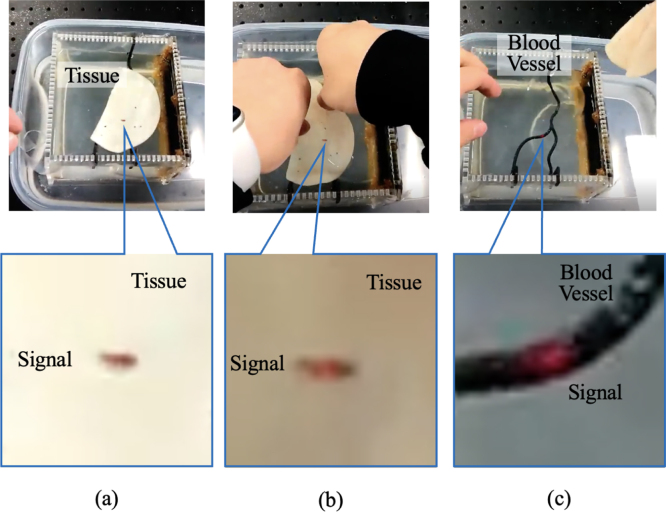
Photoacoustic signals (a) before, (b) during, and (c) after tissue was removal to correlate the location of the locked signals with the underlying vessel location.

### 3.4 Target marking validation

Fig. 9 shows photographs of the results from the target marking task. The target blood vessel was initially covered by tissue, and photoacoustic signals identified with augmented reality assistance were locked in place (Fig. 9(a)). As the tissue was removed to correlate the location of the locked signals with the underlying vessel location (Fig. 9(b)), the locked signals remained in place. After the tissue was removed (Fig. 9(c)), the physical vessel position matched the augmented reality-marked location, which confirms the ability of our system to visualize subsurface vascular structures hidden by tissue. Supplementary Video 1 shows the entire temporal progression of this validation result.

## 4 Discussion

The work presented herein introduces real-time augmented reality guidance to address laser–ultrasound alignment challenges during photoacoustic-guided surgery with freehand light delivery separated from acoustic receivers, revealing five key insights. First, through the combined use of Kalman filtering and multi-marker pose averaging, the system achieves stable tracking critical for clinical usability. Second, rendering the virtual photoacoustic image directly within the field of the imaging environment (Figs. 4(c) and 9) eliminates the need to divert attention to a separate monitor, thereby reducing cognitive load and improving hand-eye coordination (Fig. 6(a)). Third, the mean targeting error of 3.31 mm (Fig. 6(b) and Table 3), representing a 58% improvement over conventional monitor-based alignment, demonstrates the potential of augmented reality to enhance precision and workflow efficiency in photoacoustic-guided surgeries. Fourth, the total variance of laser-image intersection distribution of 12.0 mm${}^{2}$, compared to 45.9 mm${}^{2}$ without augmented reality (Table 3), shows improvement of hand motion stability around the desired laser-image intersection. Fifth, multiple desired locations throughout the image plane can be successfully targeted with augmented reality guidance (Fig. 7, Fig. 8).

The 3.31 mm mean targeting error reported in Table 3 is considered an acceptable tracking accuracy, based on previous Monte Carlo simulations demonstrating that photons can reach targets located outside the direct laser beam path due to tissue scattering [42]. While the proportion of delivered optical energy decreases as the target distance from the beam path increases [43], [44], [45], Rich et al. [23] reported that the SNR of target structures dropped from 19 when intersecting a laser beam with a custom light delivery method to 11 at a target-to-laser-beam separation distance of 3 mm, which was sufficient for imaging. However, the SNR further dropped to <7 at a target-to-laser-beam separation distance ≥6 mm. Therefore, without augmented reality guidance to achieve 3.31 mm tracking accuracy, reliable identification of critical structures during surgery will be compromised. The 16.2% “hit rate” and 7.88 mm mean targeting error obtained without augmented reality (Table 3) represent intermittent alignment success with an otherwise unacceptable mean target-to-laser-beam separation distance. With the translational and rotational calibration errors approximated as 1.26 mm 1–2°, respectively (Section 2.3) and with the 1.37 mm linear translation accuracy achieved after Kalman filtering (Section 2.4), the remaining error components (e.g., operator hand motion) apparently dominate the overall 3.31 mm tracking accuracy.

It is promising that the presented tracking accuracy was achieved despite inherent challenges associated with the small form factor (i.e., approximately 30 × 50 × 3 mm) of the laser source (Fig. 4(a)) [46]. The multi-marker design ensures detection reliability across a wide range of laser positions [47], which are frequently adjusted during photoacoustic-guided procedures to optimize the incident angle [22]. The Kalman filter further compensates for the temporary loss of visual markers by assuming continuous hand motion during operation. The adaptive adjustment of measurement noise based on marker visibility enabled the filter to dynamically balance confidence between real-time tracking accuracy and motion assumption, minimizing unrealistic jitter and abrupt pose changes. This combination of hardware and algorithmic design supports robust 6-DOF pose estimation under suboptimal visibility and mechanical constraints.

Beyond improving tracking accuracy (relative to not having augmented reality guidance), our system also enhances procedural efficiency by streaming and visualizing the virtual photoacoustic image in the original position of the physical world. The signals within the rendered virtual photoacoustic image provide confirmation of laser-plane intersection, eliminating the need to repeatedly shift attention to the ultrasound monitor. The elliptical visual element (Fig. 4(c)), shown with an intersection event in the virtual world, provides additional immediate visual feedback for laser-image alignment success. The integration of these visualization and tracking components enables accurate execution of various targeting tasks, including static center alignment (Fig. 6), continuous motion (Fig. 7), and multi-point targeting (Fig. 8). As these three tasks represent the universal baseline for photoacoustic-guided surgical maneuvers, evaluating them in isolation allows for a clear distinction between core system usability and specific alignment performance factors.

Although quantitative errors were not measured for the continuous motion and multi-point targeting tasks due to the absence of ground truth target positions, the recorded trajectories of the laser–ultrasound intersection points followed the intended motions of the users (i.e., spiral motion in Fig. 7 and multi-point targeting in Fig. 8). The intersection results for spiral motion and multi-point targeting without augmented reality guidance were not reported, because these two tasks could not be reliably executed without intuitive and real-time visual assistance, highlighting the benefit of our system for complex navigation. These observations suggest that augmented reality guidance enables users to accurately align the laser illumination area with arbitrary regions of interest on the photoacoustic imaging plane, thus facilitating optimal contrast enhancement around critical structures in the field of view [43], [48], [49], [50].

A new consideration raised by this work is the relationship between augmented reality-guided alignment and conventional signal-based alignment approaches. Signal-based methods displayed on external monitors are effective for a range of applications, particularly preclinical single-target localization with experienced operators. Augmented reality provides complementary capabilities that address specific clinical workflow constraints. By overlaying photoacoustic imaging feedback directly within the field of view of the surgeon, augmented reality eliminates the need to divert attention between the surgical site and a separate display, potentially accelerating intraoperative decision-making in time-critical scenarios. Beyond improved visualization ergonomics, augmented reality enables direct visualization of the virtual laser beam and imaging plane geometry relative to anatomy, allowing surgeons to plan optimal positioning strategies before signal acquisition.

This concept was preliminarily evaluated in a mock surgical scenario requiring subsurface vessel tracking and depth-specific target localization (Fig. 9). Although conventional photoacoustic-guided procedures require image processing (e.g., dynamic range adjustment and intensity thresholding) and mental co-registration between the image and anatomy, our initial validation simultaneously performs both of these steps. With marker-based tracking and photoacoustic signal-based alpha blending rendered via a custom shader, we successfully highlighted a hidden structure while suppressing low-amplitude background noise. In a real surgical scenario, it is conceivable that the photoacoustic signals of target structures can be directly registered to the physical anatomy in the field of view of the surgeon. This combination has the potential to streamline photoacoustic-guided surgery workflows.

While augmented reality tracking is independent of tissue optical properties, one potential limitation of our initial feasibility demonstrations herein is that the translation from observed geometric alignment to improved photoacoustic imaging performance was not investigated. Therefore, future validation with quantitative image quality metrics, followed by additional experiments with *ex vivo* tissue are planned next steps prior to *in vivo* testing and clinical translation. Future work may also include automated calibration methods that jointly optimize both the laser–holder and transducer–holder transformations [27] to enable intraoperative calibration verification if needed.

## 5 Conclusion

This paper presents the first known augmented reality photoacoustic image guidance system, intended to improve real-time laser-transducer alignment. This system reduces targeting errors by 58% relative to traditional monitor-based alignment. This research establishes augmented reality as a promising approach to address a fundamental usability challenge that has limited the adoption of photoacoustic-guided surgery with light sources separated from acoustic receivers. Our results provide a promising foundation, including a novel technical framework and the baseline performance characterization necessary for future tissue-based validation with quantitative image quality metrics. These critical next steps promise to advance this concept toward clinical application and validation.

## CRediT authorship contribution statement

**Junhao Zhang:** Writing – review & editing, Writing – original draft, Visualization, Validation, Supervision, Software, Methodology, Investigation, Formal analysis, Data curation, Conceptualization. **Cynthia Li:** Writing – original draft, Visualization, Validation, Software, Methodology, Investigation, Formal analysis, Data curation. **Muyinatu A. Lediju Bell:** Writing – review & editing, Visualization, Supervision, Resources, Project administration, Methodology, Investigation, Funding acquisition, Formal analysis, Conceptualization.

## Declaration of competing interest

The authors declare that they have no known competing financial interests or personal relationships that could have appeared to influence the work reported in this paper.

## Data Availability

Data can be made available upon reasonable request to the corresponding authors.

## Appendix A Supplementary data

Video S1 Validation of augmented reality assistance to identify photoacoustic signals from a subsurface vessel hidden by tissue. Video S1
